# Exploring the Influencing Factors of Learning Burnout: A Network Comparison in Online and Offline Environments

**DOI:** 10.3390/bs15070903

**Published:** 2025-07-03

**Authors:** Jiayao Lu, Sihang Zhu, Ranran Wang, Tour Liu

**Affiliations:** 1 Faculty of Psychology, Tianjin Normal University, Tianjin 300387, China; 15022416268@163.com (J.L.); 17399719622@163.com (S.Z.); 15955885928@163.com (R.W.); 2 Key Research Base of Humanities and Social Sciences of the Ministry of Education, Academy of Psychology and Behavior, Tianjin Normal University, Tianjin 300387, China; 3 Center of Collaborative Innovation for Assessment and Promotion of Mental Health, Tianjin Normal University, Tianjin 300387, China

**Keywords:** learning burnout, peer comparison, network analysis, network comparison

## Abstract

This study aims to explore the interrelationships among key factors influencing learning burnout, such as motivation and negative emotions (depression, anxiety, and stress) along with other factors influencing including problematic mobile phone use, nomophobia, and interactive learning, as well as whether their pathways of influence on learning burnout differ between online and offline learning contexts. Using the convenience sampling method, data from 293 college students were collected. Measurements were carried out using the Nomophobia Scale, the Problematic Mobile Phone Use Scale, the Depression Anxiety Stress Scale (DASS), the Interactive Learning Scale, the Learning Burnout Scale, and the Scale of Motivation for Activity Participation. By applying network analysis and network comparison methods, and based on the Social Comparison Theory and the Affective Socialization Heuristics Model, it was found that under the online learning condition the motivation to pursue value directly affects learning burnout. In contrast, under the offline learning condition learning motivation indirectly affects learning burnout through negative emotions. This study posits that this difference is caused by peer comparison. In a collective learning atmosphere, students’ comparison with their peers triggers negative emotions such as anxiety and stress. These negative emotions weaken the learning motivation to pursue value, ultimately resulting in an elevated level of learning burnout.

## 1. Introduction

### 1.1. Learning Burnout in Educational Contexts

In the contemporary educational landscape, learning burnout among college students has emerged as a highly salient concern, wielding a non-trivial negative influence on students’ academic achievements, as well as their physical and mental well-being (Fredricks et al., 2004). Learning burnout is defined as the state in which students encounter emotional lassitude, loss of interest, and diminution of motivation and self-confidence during the learning journey, which is frequently accompanied by physical and mental fatigue and a downturn in academic performance (Maslach & Jackson, 1981). With the exponential development of informatization and the incessant evolution of the economic and employment situation, college students are confronted with unprecedented opportunities and challenges. Correspondingly, the issue of learning burnout has become increasingly acute. A survey by the American Psychological Association reveals that over 30% of college students have endured severe learning burnout. This not only precipitates a marked deterioration in their academic performance but also gives rise to a substantial increase in emotional issues (American Psychological Association, 2018). Moreover, according to a 2022 survey by the American College Health Association (ACHA), four out of five college students report experiencing burnout during their undergraduate experience (American College Health Association, 2022).

The development of informatization and the changes in the economic and employment situation have increased the opportunities and challenges that college students need to deal with. Against this educational backdrop, the learning burnout of college students has become a problem that cannot be overlooked. Offline learning refers to a teaching mode in which teachers and students complete knowledge transfer through face-to-face interaction, physical tools, and synchronous activities in a physical space (Graham, 2019). With the advancement of technology and the popularization of the Internet, online learning, which has the advantages of low cost, convenience, and rich resources, is attracting more and more attention globally as a new type of learning mode. Blended learning (BL), which combines online resources with offline interactions, is generally believed to be able to balance flexibility and engagement (Feng et al., 2018). Therefore, the situation of learning burnout under different learning conditions has received great attention. Identifying the paths that affect learning burnout and helping college students find ways to alleviate it are of great significance for stimulating students’ enthusiasm for learning and cultivating high-quality talents.

Learning burnout is defined as a negative psychological state that occurs during the learning process, specifically manifested as a significant decline in learning motivation and efficiency, as well as a negative expectation towards future learning (Maslach & Jackson, 1981). This phenomenon not only has a negative impact on students’ academic performance but may also have adverse effects on their physical and mental health, and it is closely related to various factors such as motivation, learning pressure, and learning methods (Fredricks et al., 2004).

Motivation, as the internal driving force that propels students to learn, plays a crucial role in the learning process (Ryan & Deci, 2000b). It can not only stimulate students’ learning behaviors but also maintain and regulate the intensity and duration of learning. Motivation can be divided into intrinsic motivation and extrinsic motivation. Intrinsic motivation stems from students’ interest in and love for the learning content itself, while extrinsic motivation comes from external rewards, recognition, or pressure (Miller et al., 1988).

Numerous studies have shown that there is a close relationship between motivation and learning burnout (Pekrun et al., 2009). Students with high intrinsic motivation tend to be passionate about learning, actively participate in learning activities, and are more likely to experience a sense of achievement and satisfaction in learning, thus reducing the probability of learning burnout (Ryan & Deci, 2000b). A study on college students found that students with a strong interest in their majors had significantly lower levels of learning burnout than those without interest (Schiefele, 1991). Although extrinsic motivation can, to some extent, stimulate students’ learning behaviors, excessive reliance on external rewards may lead to a decline in students’ interest in learning itself (Deci et al., 1999). In addition, the achievement goal theory suggests that students’ achievement goal orientations also affect learning burnout (Elliot & McGregor, 2001). The achievement goal theory, which is closely linked to motivation, has a significant impact on learning burnout. Students’ achievement goal orientations significantly influence their learning burnout levels by affecting their motivation. Students with a mastery goal orientation are driven by intrinsic motivation, focusing on enhancing their abilities and acquiring knowledge. They value the learning process, which makes them more persistent in facing difficulties and helps them maintain a lower level of learning burnout. Conversely, students with a performance goal orientation are often motivated by extrinsic factors, such as external recognition and rewards. They focus on comparing themselves with others and obtaining external evaluations. When facing setbacks, they are prone to experiencing negative emotions such as anxiety and frustration. These emotions can weaken their intrinsic motivation to pursue value, ultimately leading to elevated levels of learning burnout (Pintrich, 2000; Ryan & Deci, 2000a). Research has shown that students with a performance goal orientation are more likely to experience learning burnout than those with a mastery goal orientation (Midgley et al., 2001). In academic settings, students with a performance goal orientation reported higher levels of learning burnout symptoms compared to those with a mastery goal orientation. This supports the idea that the achievement goal theory provides a framework for understanding how students’ motivational orientations can shape their experiences of learning burnout.

Emotions, which represent individuals’ subjective experiences of objective matters, exert a profound influence on the learning process and the manifestation of learning burnout (Pekrun, 2006). Positive emotions like joy and interest can effectively stimulate students’ learning motivation. They enhance learning efficiency, boost students’ enthusiasm and initiative in learning, and play a crucial role in reducing the likelihood of learning burnout (Fredrickson, 1998). Mirvis and Csikszentmihalyi (1991) pointed out that when students experience a sense of happiness and fulfillment during the learning process they are more inclined to invest time and energy and actively explore new knowledge. This proactive engagement significantly decreases the probability of learning burnout. On the contrary, negative emotions such as depression, anxiety, and stress have detrimental effects on learning and are key factors contributing to learning burnout (Pekrun et al., 2006).

Negative emotions, including depression, anxiety, and stress, collectively contribute to learning burnout.

Depression can cause students to fall into a state of low mood, leading them to lose interest in learning and weaken their learning motivation. Therefore, students may adopt negative attitudes and behaviors toward learning, thereby increasing the risk of academic burnout (Clark & Watson, 1991). Although the study conducted by Clark and Watson in 1991 was based on a mixed sample, the tripartite model they proposed has been validated in the college student population. For example, Joiner’s (1996) study supported this model, confirming that depression and anxiety share a common negative affective component while also having unique symptom dimensions, which provides a basis for the application of this model in research on college students.

Anxiety can interfere with students’ attention and limit their thinking abilities, thus having a negative impact on learning outcomes. If students remain in an anxious state for a long time, they may develop a tendency to fear and avoid learning, ultimately leading to academic burnout (Sarason, 1984). Sarason’s (1984) study on college students showed that the mechanism of academic stress is particularly prominent in the higher education environment, which is consistent with the characteristics of the college student sample in this study. Emotions may have mediating or moderating effects on learning burnout (Frenzel et al., 2007).

Previous studies have shown that negative emotions may indirectly affect learning burnout by influencing students’ self-efficacy and learning motivation (Bandura, 1977). When students experience negative emotions their confidence in their learning abilities may decline and their learning motivation may weaken, making them more prone to learning burnout. In addition, emotions may also moderate the relationship between other factors and learning burnout. For example, in high-stress situations, negative emotions may enhance the impact of stress on learning burnout (Pekrun, 2006).

### 1.2. Theoretical Framework: Social Comparison and Affective Processes

In real-life situations, people usually define their own social characteristics (such as abilities, intelligence, etc.) by comparing themselves with those around them. The acquisition of such meaning is achieved in a comparative social environment, rather than based on purely objective criteria (Xing & Yu, 2005). Festinger named this phenomenon social comparison. Social comparison, also known as interpersonal comparison, refers to the process of comparing an individual’s situation and status (such as abilities, opinions, physical health, etc.) with those of others. The core idea of this theory is that people tend to obtain information about their social status by comparing themselves with others. This comparison process has been divided by later generations into upward comparison (comparing with more excellent people), downward comparison (comparing with less excellent people), and parallel comparison (comparing with people at the same level as oneself). Over time, the social comparison theory has undergone significant development and expansion. Research shows that social comparison is not just a simple self-evaluation process, but a complex psychological phenomenon involving multiple motives, strategies, and situational factors (Wood, 1989). For example, people may engage in social comparison because they want to enhance their self-esteem, seek information, or improve their situation. In addition, the process of social comparison is not one-way, but two-way, and can take various forms to meet different goals of individuals.

Social comparison theory posits that individuals evaluate their capabilities and values by comparing themselves with others, particularly through the following three modes: upward (superior others), downward (inferior others), and parallel comparisons (similar others) (Festinger, 1954; Wood, 1989). Peers, as the objects that individuals frequently interact with in study and life, and their performances, achievements, and attitudes naturally serve as important references for individuals to make comparisons (Festinger, 1954).

Peer comparison, based on the social comparison theory, refers to the process in which individuals compare their abilities, achievements, or other characteristics with those of others to assess their own position and value. This comparison process can be driven by various motives, including self-improvement, information-seeking, and self-esteem enhancement (Taylor & Lobel, 1989). Peer comparison is not limited to direct comparisons between individuals but may also involve comparisons in a broader social context, such as comparing with friends, classmates, or other members of a social group (Lubbers et al., 2009).

In specific social environments the motives and outcomes of peer comparison may vary. For example, in threatening situations, individuals may tend to make downward comparisons to boost their self-esteem. In daily life, however, individuals may be more likely to make upward comparisons to seek opportunities for self-improvement (Ding, 2018). In addition, peer comparison can also be influenced by individual traits. For instance, adolescents with higher self-control abilities may be more prone to engage in risky behaviors, indicating that peer comparison is related to individuals’ internal traits.

Previous studies have shown that peer relationships have a significant impact on students’ learning experiences and psychological states. A good peer relationship can help relieve students’ depression and anxiety and improve their social–cognitive abilities. On the contrary, poor peer relationships may lead to emotional problems such as loneliness and anxiety, which in turn affect learning engagement and outcomes (Altermatt & Pomerantz, 2005). In the context of certain learning pressures, peer comparison may give rise to negative peer relationships. This negativity does not necessarily manifest as poor interpersonal relationships among peers, but rather as psychological stress and comparison, thus influencing the learning state and motivation. It can be seen that peer comparison is one of the important factors contributing to learning burnout. When students feel that they are at a disadvantage among their peers, they may experience frustration, inferiority, and anxiety, resulting in a decline in learning motivation, a decrease in learning interest, and a deterioration of learning outcomes. In the context of this study examining online and offline learning environments, peer comparison operates through different mechanisms. In online environments, the lack of real-time peer interaction reduces opportunities for spontaneous upward comparison (e.g., observing classmates’ performance), prompting students to rely on intrinsic value pursuit (e.g., “I want to acquire knowledge”) rather than external benchmarks (Ding, 2018; Ryan & Deci, 2000b). In offline environments, peer proximity enhances two-way social evaluation. Upward comparison may trigger anxiety-driven self-doubt, while downward comparison, although promoting temporary self-enhancement, is detrimental to students’ long-term development. Therefore, peer comparison can explain different pathways of learning burnout. In online environments, value-driven motivation directly reduces burnout through autonomous goal orientation. In offline environments, negative emotions triggered by peer comparison mediate the relationship between motivation and burnout.

Research on the Affective Socialization Heuristic Model mainly focuses on the process of affective socialization and its impact on children’s development (Eisenberg et al., 1998). Eisenberg (2020) further deepened this theory by describing emotion-related socialization in classrooms. The relationship between the socialization agent and the outcome may be moderated by emotions, others, the classroom environment, and student characteristics. In particular, the peer-comparison based Affective Socialization Heuristic Model indicates that peer comparison not only affects an individual’s cognitive evaluation but also triggers specific emotional responses and behavioral choices. In offline environments, peer comparison may cause individuals to have positive or negative emotional experiences, which in turn affect their learning attitudes and behaviors.

### 1.3. Selection of Extraneous Variables

Many previous studies have extensively explored the influencing factors of learning burnout (Pekrun et al., 2006; Ryan & Deci, 2000b). To ensure the accuracy and reliability of the research results, this study also includes some variables that may affect learning burnout in the research scope, such as nomophobia (NMP), problematic mobile phone use (PMPU), and interactive learning, but they are not used as key analysis variables. The aim is to more comprehensively control potential interfering factors and make the research results more persuasive.

NMP and PMPU are the extraneous variables included in this study. NMP refers to negative emotions such as anxiety and restlessness that individuals experience when they cannot use their mobile phones (Safaria et al., 2024), which may confound emotional states (e.g., stress) and reduce learning engagement, thereby indirectly influencing burnout. PMPU involves issues such as excessive dependence on mobile phones and the inability to control mobile phone use behaviors, which can fragment attention and diminish academic efficiency, potentially masking the direct relationship between motivation and burnout (Billieux et al., 2015). Although these variables are not the core of the study, controlling them is necessary to isolate the independent effects of key variables such as motivation and emotion on learning burnout. For example, PMPU has been proven to lead to academic performance decline by depleting attention (Billieux et al., 2015), while NMP may amplify stress responses that overlap with burnout symptoms (Safaria et al., 2024). By including these confounding factors, this study enhances the internal validity of network analysis, ensuring that the observed associations reflect real psychological mechanisms rather than technological or behavioral interference (Epskamp et al., 2018).

Additionally, the study also includes interactive learning—defined as collaborative activities in the learning process (Dillenbourg, 1999)—to assess its potential indirect effects. For instance, peer interaction in offline environments may alleviate loneliness (Gillett-Swan, 2017), whereas online interaction lacks immediacy and may influence burnout differently. By controlling these variables, the study clarifies the unique pathways through which core factors (such as motivation and peer comparison) operate in different learning environments.

### 1.4. The Importance of Network Analysis Methods

Network analysis is a method of exploring the interactions and internal structures among different components of a specific psychological construct or related behaviors through visualization (Borsboom & Cramer, 2013). In network analysis, the network structure is usually analyzed based on Graph Theory, and the specific relationships among things are presented through visualized network diagrams, which can intuitively display complex problems and help researchers better understand the relationships among variables and their impacts on the whole. In current psychological research, with the rise in high-dimensional big data, the number of variables involved continues to increase. Network analysis can reveal the correlation patterns among multiple variables simultaneously. Nowadays, in fields such as clinical psychology, personality psychology, and social psychology, network analysis is widely used to supplement or revise the conclusions of previous models and discover patterns that are difficult to identify with traditional analysis methods (Hevey, 2018).

In this study, since multiple variables are involved, such as nomophobia, problematic mobile phone use, depression, anxiety, stress, interactive learning, learning burnout, the motivation to enjoy and relax, the motivation to pursue value, etc., network analysis methods can effectively handle these variables, reveal the complex relationships among them, and provide a powerful tool for a deeper understanding of the influencing factors of learning burnout. By constructing a network model, the interactions among various variables can be visually displayed, and the key nodes and important relationships can be identified, thus providing support for the verification of research hypotheses and the drawing of conclusions.

### 1.5. Research Questions and Hypotheses

Based on the literature review and the characteristics of online and offline learning, online learning lacks peer comparison and peer pressure and is more independent, while offline learning takes place in a collective environment, with peer comparison and peer pressure, and is more interactive. Under such differences, students’ learning motivations also vary. The learning motivation of students in online learning may be more inclined towards their own internal drive and the pursuit of self-value (for example, “I want to acquire more knowledge.”). The stronger the motivation, the lower the level of learning burnout may be. However, the learning motivation of students in offline learning may not only include the goal of pursuing self-value but also be influenced by peer comparison (for example, “I can’t be worse than others”). This kind of comparison can bring a certain degree of negative emotions, which in turn affects the learning motivation. Higher negative emotions may be accompanied by lower learning motivation, resulting in a higher level of learning burnout.

Based on the literature review and theoretical framework, we propose the following hypotheses:

**H1.**  *In online learning environments, the motivation to pursue value directly reduces learning burnout.*

**H2.**  *In offline learning environments, peer comparison triggers negative emotions (e.g., anxiety), which mediate the relationship between motivation and learning burnout.*

**H3.**  *The network structure of burnout factors differs significantly between online and offline conditions, with peer comparison driving these differences.*

With the increasing complexity and diversification of the educational environment, students are facing more opportunities for peer comparison in their studies. Peer comparison is ubiquitous, whether in classroom discussions, group cooperative learning, or extracurricular activities. Understanding the influence mechanism of peer comparison on learning burnout can help educators develop more targeted intervention strategies. By guiding students to establish correct comparison concepts and build positive peer relationships and by providing personalized learning support, the negative impact of peer comparison can be reduced and students’ learning effectiveness and mental health levels can be improved. Therefore, exploring the relationship between peer comparison and learning burnout has important practical significance.

This study will contribute to a deeper understanding of the formation mechanism of learning burnout and provide strong guidance for educational practice.

## 2. Materials and Methods

### 2.1. Participants

A convenience sampling method was adopted. The same group of participants completed two questionnaires distributed via the Wen Juan Xing platform at two time points, with a six-month interval between surveys. The first survey was conducted during an online learning period, with 638 participants initially enrolled (146 males and 492 females; mean age = 20.85 ± 2.00 years). Six months later, the same participants were invited to complete the second survey during an offline learning period. Of the original cohort, 403 participants (73 males and 330 females; mean age = 21.18 ± 1.99 years) responded to the second survey. After removing abnormal responses and pairing valid data from both surveys, 293 participants who completed both surveys were retained for final analysis. 

The two surveys were conducted in the second week after the start of the spring semester (during the online learning period) and the second week after the start of the autumn semester (during the offline learning period), respectively, with an interval of six months. All participants completed the surveys in a fixed order: online first and then offline. To minimize the interference of academic stressors (e.g., final exams, project deadlines), data collection was scheduled during periods of relatively light academic workload at the beginning of each semester.

This study was approved by the Ethics Review Committee of Tianjin Normal University. Before data collection, all participants signed an electronic informed consent form, confirming that they had fully understood the purpose, procedures, and potential risks/benefits of the study, and voluntarily agreed to participate. To avoid response bias, specific research hypotheses such as “learning burnout” and “peer comparison” were not disclosed in the informed consent form, but participants were promised a detailed explanation after data collection was completed. The study adheres to the ethical guidelines of the Helsinki Declaration throughout.

### 2.2. Research Tools

The Nomophobia Scale, the Problematic Mobile Phone Use Scale, the Depression Anxiety Stress Scale (DASS-21), the Interactive Learning Scale, the Learning Burnout Scale, and the Scale of Motivation for Activity Participation were all used.

#### 2.2.1. Learning Burnout Scale

The Maslach Burnout Inventory—Student Survey (MBI-SS) was used to assess learning burnout (Schaufeli et al., 2002). This 16-item scale is composed of the following three dimensions: exhaustion (5 items; e.g., “I feel emotionally drained by my studies”), cynicism (4 items; e.g., “I doubt the significance of my coursework”), and reduced efficacy (6 reverse-scored items; e.g., “I can effectively solve academic problems”). Responses were recorded on a 7-point frequency scale (0 = never to 6 = always), with higher scores indicating greater burnout. The original validation demonstrated cross-cultural reliability (Cronbach’s α = 0.65–0.86) and structural validity (CFI ≥ 0.90 and RMSEA < 0.08; (Schaufeli et al., 2002)).

#### 2.2.2. Motivational Scale

The Hedonic and Eudaimonic Motives for Activities–Revised (HEMA-R) scale developed by Huta and Waterman (2014) was used, which contains 10 items. This scale was validated through cross-sample research by Giuntoli et al. (2021) (CFI = 0.974 and RMSEA = 0.075), and the eudaimonic orientation was positively correlated with adaptive coping strategies, while the relaxation orientation was positively correlated with avoidant coping strategies. It takes the form of the following three-factor structure: Eudaimonic orientation (5 items): Measures the motivation to pursue self-growth and value realization (example item: “Seeking to fulfill my potential”). Pleasure orientation (3 items): Measures the motivation to pursue happiness and enjoyment (example item: “Seeking fun”). Relaxation orientation (2 items): Measures the motivation to pursue comfort and stress avoidance (example item: “Seeking relaxation”). The scale uses a 7-point Likert scale (1 = “Completely inconsistent” and 7 = “Highly consistent”). Confirmatory factor analysis (CFA) supports the three-factor model (Giuntoli et al., 2021) with excellent fit indices of χ^2^ (32) = 115.7, CFI = 0.974, TLI = 0.964, RMSEA = 0.075 (90% CI: 0.061–0.090), and SRMR = 0.060, and all items have standardized factor loadings > 0.50.

#### 2.2.3. Nomophobia Scale

The Nomophobia Scale (NMP) was developed by Yildirim and Correia (2015), and its Chinese version (NMP-C) was revised by Ren et al. (2020) to assess fear of being without a mobile phone. The NMP-C consists of 16 items and includes 4 dimensions: Fear of inability to access information, fear of losing convenience, fear of losing contact, and fear of losing network connection. It uses a 7-point Likert scale, ranging from “1 = Completely inconsistent” to “7 = Completely consistent”. In this study, the Cronbach’s alpha coefficient of the total scale was 0.95.

#### 2.2.4. Problematic Mobile Phone Use Scale

The Problematic Mobile Phone Use Scale—Very Short Version (PMPUS-VS) developed by Zhou et al. (2023) was used, which contains 4 items. Confirmatory factor analysis supports a single-factor structure for this scale (χ^2^/*df* = 2.48, CFI = 0.99, and RMSEA = 0.07), and it has a significant positive correlation with the total score of the Mobile Phone Addiction Tendency Scale (MPATS) for college students (*r* = 0.82). The measurement dimension is withdrawal behavior (4 items), which evaluates negative physical and psychological reactions when unable to use a mobile phone (example item: “I feel uneasy if I don’t use my mobile phone for a period of time”). The scale uses a 5-point Likert scale (1 = “Highly inconsistent” and 5 = “Highly consistent”), and all items have standardized factor loadings > 0.50.

#### 2.2.5. DASS-21 Scale

The Depression Anxiety Stress Scales-21 (DASS-21; Lovibond & Lovibond, 1995) assessed 3 domains with 7 items each, using a 4-point severity scale (0 = did not apply to me to 3 = applied to me very much). Subscale scores were doubled to align with DASS-42 norms. CFA supported the tripartite structure (*χ*^2^/*df* = 2.89, CFI = 0.92, and RMSEA = 0.07), with Cronbach’s α coefficients of 0.89 (depression), 0.84 (anxiety), and 0.86 (stress). Concurrent validity was demonstrated through strong correlations with PHQ-9 scores (*r* = 0.79 for depression).

#### 2.2.6. Interactive Learning Scale

The Interactive Learning Scale (Kuo et al., 2014) was used in this study. It has the following three dimensions: learner–learner interaction (8 items, α = 0.94), learner–instructor interaction (6 items, α = 0.83), and learner–content interaction (3 items, α = 0.92). All use a 5-point Likert scale. The scale shows good reliability and validity, confirmed by expert content validity testing (CVR) and pretesting.

### 2.3. Research Methods

Data were pre-processed using SPSS 26.0, and descriptive statistics and correlation analyses were performed. Subsequently, R (4.3.1) was used to write the codes required for network analysis. Based on the longitudinal data collected from the follow-up survey, attempts were made to conduct network analysis. The “bootnet” package was used for network construction and stability testing; the “qgraph” package was used for network visualization and calculation of centrality indices of network nodes; the “mgm” package was used to estimate the predictability of nodes; the principal component analysis method was used to verify the reliability of the network structure; and finally, the “NetworkComparisonTest” package was used for network comparison.

#### 2.3.1. The Concept and Models of Network Analysis

A network consists of a series of nodes and edges between the nodes. Nodes represent variables, and the edges between nodes represent the relationships between variables (Hevey, 2018). For example, in the core symptom network of bulimia nervosa, anxiety, and depression constructed by previous researchers, variables such as the behaviors of bulimia nervosa and the core symptoms of anxiety and depression represent the nodes in the network, while the positive and negative relationships between these nodes are the edges (Levinson et al., 2017). In a psychological network, nodes represent various psychological variables, such as attitudes, cognitions, emotions, symptoms, and behaviors. The edges represent unknown statistical relationships that can be estimated from the data, such as correlations and predictive relationships. The selection of nodes depends on the data type, and these data types provide the most appropriate and useful understanding for the problem to be solved. Centrality is usually used to understand the centrality of a node in the network. Generally, there are the following three common indicators: Strength, Closeness, and Betweenness. Edges can represent different types of relationships, such as the comorbidity of psychological symptoms and the correlations between attitudes. The characteristics of edges are generally understood and described through the following three aspects: Weight, Sign, and Direction. Weight refers to the degree of closeness between the nodes connected by the edge in the network. The thicker the edge, the closer the relationship between the two nodes. The Sign refers to the color of the edge. Usually, green or blue edges represent positive relationships, and red edges represent negative relationships. Direction is an indicator used to describe whether there is a causal relationship in the network. If there is a causal relationship between nodes, and in the network diagram an arrow on the edge indicates a causal relationship between the connected nodes, then the node being pointed to is the result and the node that points is the cause.

A network that only considers whether there is a connection between nodes is called an unweighted association network. Since an unweighted network can only describe the relationships between nodes through binary classification, it usually cannot fully depict the characteristics of a system. When conducting research in psychology using continuous indicators, it is not enough to only focus on whether there is an association between observed variables: the degree of these associations also needs to be considered (Newman, 2004). In this case, a weighted correlation network with a way to represent the strength of connections is more suitable for depicting the relationships between observed variables, because in this network model the connections represent the strength of the relationships between nodes. Due to different model fitting methods, by adding the consideration of the influence of the weighted values of connections based on the indicators of unweighted correlation networks, weighted correlation network indicators can be obtained to describe each node and the entire network structure (Barrat et al., 2004).

The Gaussian graphical model based on partial correlation analysis is applicable to cross-sectional data where all variables are continuous variables. However, network analysis methods for other data types are constantly being proposed. For example, for binary variable data the network analysis method based on the Ising model can be adopted. Logistic regression is used to calculate the strength of the connections between nodes, and similar penalty factors can be used to simplify the network. In addition, for data that contains both categorical and continuous variables Haslbeck and Waldorp (2015) proposed the mixed graphical model to establish the corresponding network analysis method. For longitudinal data, researchers have proposed an analysis method, that is, using the regression coefficients between variables to represent the values of the connections between nodes (Epskamp et al., 2018). In longitudinal network models, since the measurement of variables has a time-series order, mutual prediction can be carried out. For different types of longitudinal data, researchers have gradually developed other types of network models. For example, for time-series data of a single observed variable, there are the vector autoregressive model (Bringmann et al., 2013) and the principal component autoregressive model (Pan, 2017). For multiple observed variables, there are the multilevel autoregressive network model (Liu, 2021) and the cross-lagged network model for a small number of measurement time points (Rhemtulla et al., 2017).

#### 2.3.2. Indicators Related to Network Analysis

Network analysis presents the characteristics and information of complex systems in the form of networks. When studying complex systems, people can transform problems into those related to the structure or features of the corresponding network diagram and conduct descriptive analysis. Among them, the centrality of nodes has always been a crucial issue, representing the number, strength, and tightness of connections between a node and other nodes (Freeman, 1978). There are three commonly used centrality indicators, which are as follows: strength centrality, betweenness centrality, and closeness centrality.

(1) Strength Centrality

Strength centrality represents the total weight of connections between a node and other nodes, reflecting its direct influence on the network. The higher the strength value, the more central the node is to the network. For example, if “depressive mood” has a high strength centrality, it indicates a close association with learning burnout and other factors, making it a potential key target for intervention.

(2) Betweenness Centrality

Betweenness centrality measures a node’s ability to act as a “bridge,” i.e., the frequency with which it lies on the shortest paths between other nodes. Nodes with high betweenness centrality (such as “learning burnout”) may indirectly influence the entire network by regulating the interactions between other variables, revealing potential mediating mechanisms.

(3) Closeness Centrality

Closeness centrality reflects the average distance between a node and all other nodes in the network. The shorter the distance, the more likely the node is to rapidly influence the entire network. For instance, if “stress” has high closeness centrality, it signifies that it can quickly act on other variables through direct or indirect paths, highlighting its importance in the dynamic process of learning burnout.

In this study, the above indicators were used in combination with network visualization to compare the differences in the network structures of influencing factors of learning burnout between online and offline environments. For example, core driving factors were identified through strength centrality and indirect paths were explored through betweenness centrality, thereby revealing the heterogeneity in the formation mechanisms of learning burnout under different learning environments.

#### 2.3.3. Indicators Related to Network Comparison 

Network comparison aims to explore the differences and similarities between different networks. Van Borkulo et al. (2023) proposed to conduct network comparisons based on a permutation test, namely the Network Comparison Test (NCT). NCT assesses differences from two aspects: global invariance (whether overall network structures are the same) and local invariance (whether specific edges or node centralities differ). This method helps reveal changes in variable relationships under different learning conditions.

In network comparison, the main indicators are as follows:

I. Global Invariance Indicators
  (1) Network structure invariance:
    Network structure invariance is assessed by the maximum absolute difference of corresponding edge weights.
  (2) Network global strength invariance:
    Network global strength invariance is assessed by the sum of absolute values of all edge weights.
II. Local Invariance Indicators
  (1) Edge strength invariance:
    Edge strength invariance compares edge weights of the two networks.
  (2) Differences in node centrality indices:
    Differences in node centrality indices compare node centralities of the two networks.

These indicators help to analyze whether there are differences in the associations among learning burnout factors and their impact paths under different learning conditions.

## 3. Results

### 3.1. Common Method Bias Test

In this study, the participants filled out the scales by self-reporting, which is likely to lead to common method bias. Therefore, Harman’s single-factor test method was used for the test. The results showed that in online learning, there were three factors with eigenvalues greater than 1, and the variance explained by the largest factor was 37.986%, which was less than the critical criterion of 40%. In offline learning, there were also three factors with eigenvalues greater than 1, and the variance explained by the largest factor was 39.842%, less than the 40% critical criterion. This indicates that there is no serious common method bias problem in this study.

### 3.2. Descriptive Statistics and Correlation Analysis

**Figure 1:**
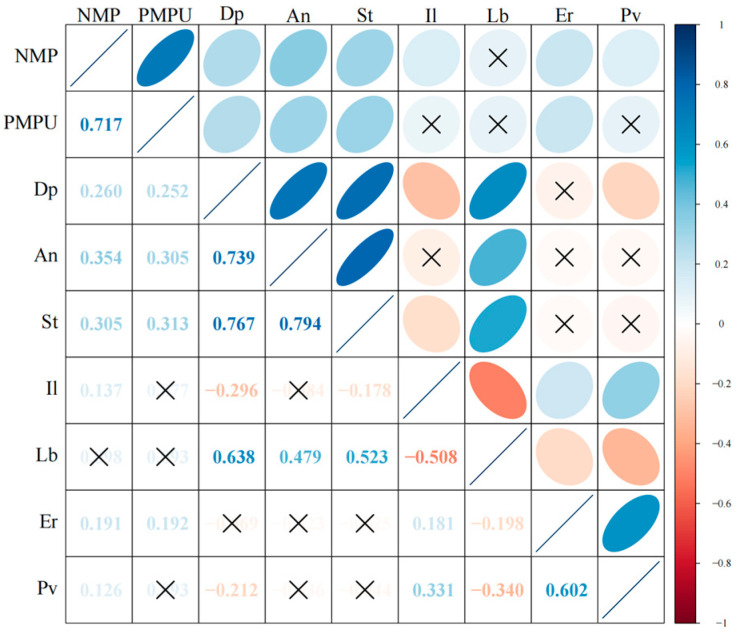
Correlation matrix of the influencing factors of learning burnout under online learning environment.

**Figure 2:**
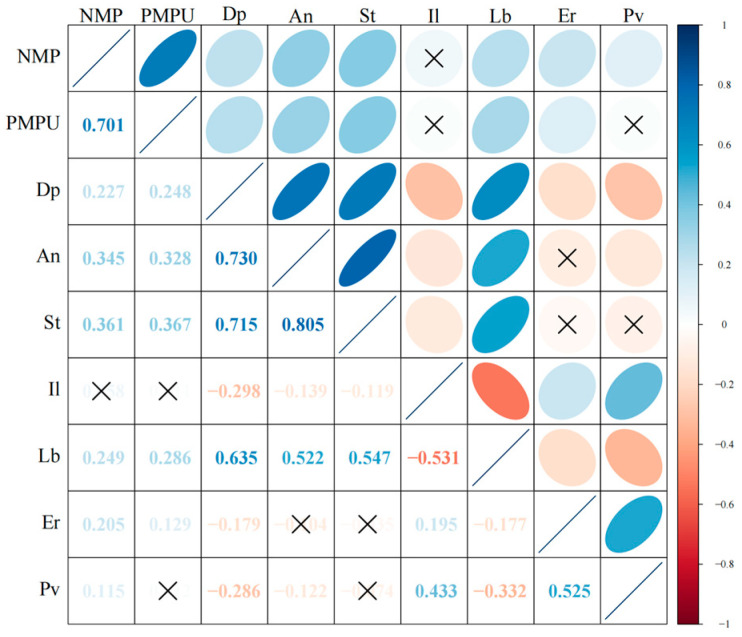
Correlation matrix of the influencing factors of learning burnout under offline learning environment.

Table 1 presents the descriptive statistical results of learning burnout and its influencing factors under online and offline learning conditions. From this, it can be preliminarily known that there are no significant differences among the influencing factors under the two conditions. Figure 1 shows the correlation matrix among learning burnout and its influencing factors under online learning conditions, while Figure 2 presents the correlation matrix among the influencing factors of learning burnout under offline learning conditions. In the matrix, the correlation coefficients among various factors are shown in the lower-left corner, and the upper-right corner visually displays the correlation relationships among the factors. The color and direction of the ellipses indicate the direction of the correlation relationships (blue, from top-right to bottom-left corresponds to a positive correlation; red, from top-left to bottom-right corresponds to a negative correlation). The color depth and shape of the ellipses represent the strength of the correlation relationships (where a darker color and a flatter ellipse correspond to a stronger correlation). And “×” indicates the *p* > 0.05 corresponding to the correlation coefficient, that is, the correlation relationship between the factors is not significant.

The results show that there are significant correlation relationships among most factors. Overall, there are more correlations among the factors in offline learning. In online learning the degree of positive correlation ranges from 0.126 to 0.794, and the degree of negative correlation ranges from −0.508 to −0.198. In offline learning the degree of positive correlation ranges from 0.115 to 0.805, and the degree of negative correlation ranges from −0.531 to −0.119. There are differences in the correlation degrees among various factors under the two conditions, but further exploration is needed to determine whether there are statistical differences.

In the online environment, variables with higher positive correlation ranges were concentrated in technology use scenarios (e.g., the association between mobile phone dependence and anxiety, *r* = 0.794), reflecting that the deep embedding of digital tools in learning may amplify specific psychological risks. In negative correlations, the stronger negative association between motivation and burnout (−0.508 to −0.198) suggests the protective effect of intrinsic motivation on autonomous learning, while the weak negative association of some interaction-related variables (e.g., interactive learning and burnout, *r* = −0.119, *p* = 0.12) may indicate that online interaction forms fail to fully exert stress-relieving effects. In the offline environment, the high-end value (0.805) of the positive correlation range appeared in the association between interactive learning and motivation, indicating that face-to-face collaboration significantly activates learning motivation; the positive correlation between depression and learning burnout (*r* = 0.28, *p* < 0.001) was stronger than that in the online environment, indicating that offline social comparison amplifies emotional effects. The positive correlation between stress and anxiety was numerically slightly higher in offline settings (*r* = 0.682) than in online settings (*r* = 0.638), which is possibly related to the immediate pressure of offline evaluations.

The differences in the distribution of correlation coefficients under different conditions reveal the influence of the social attributes of the learning environment on variable relationships. In the online environment, the technology-mediated learning model leads to a more prominent “individual psychology-behavior” pathway (e.g., motivation directly affects burnout); whereas in the offline environment, interpersonal interaction triggers a chain reaction of “social comparison-emotion-burnout”, making the associations between emotional variables closer.

### 3.3. Difference Test

A paired samples *t*-test was conducted on the scores of learning burnout and its various influencing factors under the following two different conditions: online learning and offline learning. As shown in Table 2, there were no significant differences among the various influencing factors.

### 3.4. Network Analysis

#### 3.4.1. Network Structure Estimation

**Figure 3:**
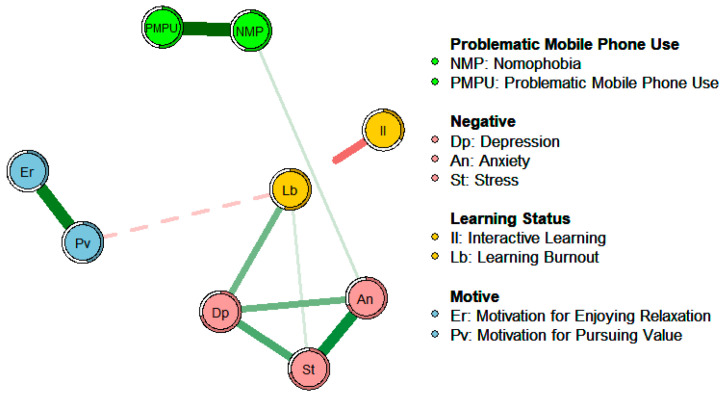
The network of influencing factors of learning burnout under online learning environment.

**Figure 4:**
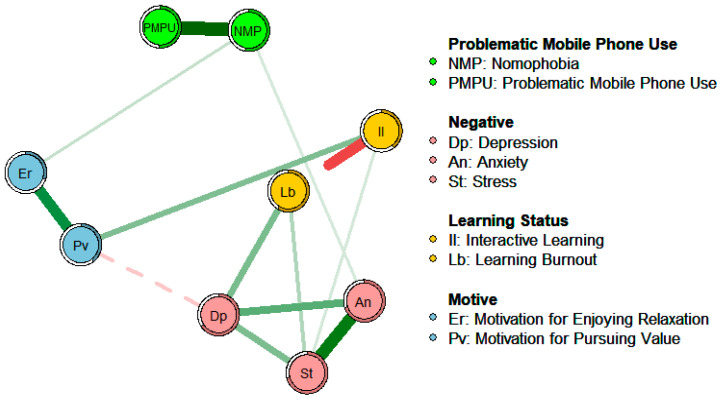
The network of influencing factors of learning burnout in the offline learning environment.

In order to explore the differences in the associations and influence paths among the influencing factors under online and offline learning conditions, networks of the influencing factors of learning burnout in online and offline learning were constructed. In the process of constructing the networks, to ensure that the network results have good reproducibility, this study adopted a regularization method. Specifically, the EBICglasso algorithm was used to introduce a penalty factor, which can compress smaller partial correlation coefficients to 0. This operation actually removes the unimportant, spurious, and weakly partially correlated connections in the network, thus obtaining a sparser network with a simpler structure and easier interpretation. Based on previous research experience (Jones et al., 2018), the sparsity parameter in this study was set to 0.3. At the same time, “threshold = TRUE” was set to simplify the network for easier observation. Additionally, the average layout function in the qgraph package was used to determine the layout of each node in the network, enabling an intuitive visual comparison of the network structures of the two groups, as shown in Figure 3 and Figure 4. Among them, the color and solid/dashed state of the connections in the network represent the direction of the partial correlation relationship (green and solid lines correspond to positive partial correlations, while red and dashed lines correspond to negative partial correlations). The thickness of the connections indicates the strength of the partial correlation relationship (the thicker the connection, the stronger the corresponding partial correlation). The rings around the nodes represent the variance of the factors represented by the nodes, and the shaded areas show the proportion of the variance of each factor that can be explained by the variables connected to it, displaying predictability.

As can be known from the results of the network analysis, under the online learning environment the motivation to pursue value has a significant negative correlation with learning burnout (*p* < 0.001, *r* = −0.15 ), which is the direct impact of motivation on learning burnout. Under the offline learning condition, the motivation to pursue value has a significant negative correlation with depressive mood (*p* < 0.001, *r* = −0.14), and depressive mood has a significant positive correlation with learning burnout (*p* < 0.001, *r* = 0.28), which is the indirect impact of motivation on learning burnout.

This result preliminarily verifies the new theoretical hypothesis proposed based on the research achievements and deficiencies of predecessors, that is, under the two different conditions of online learning and offline learning there are differences in the influencing factors of learning burnout. Under the online learning condition, the motivation to pursue value directly affects learning burnout. However, in offline learning, due to the influence of peer comparison, learning motivation mainly affects learning burnout indirectly through negative emotions.

#### 3.4.2. Network Inference Analysis

**Figure 5:**
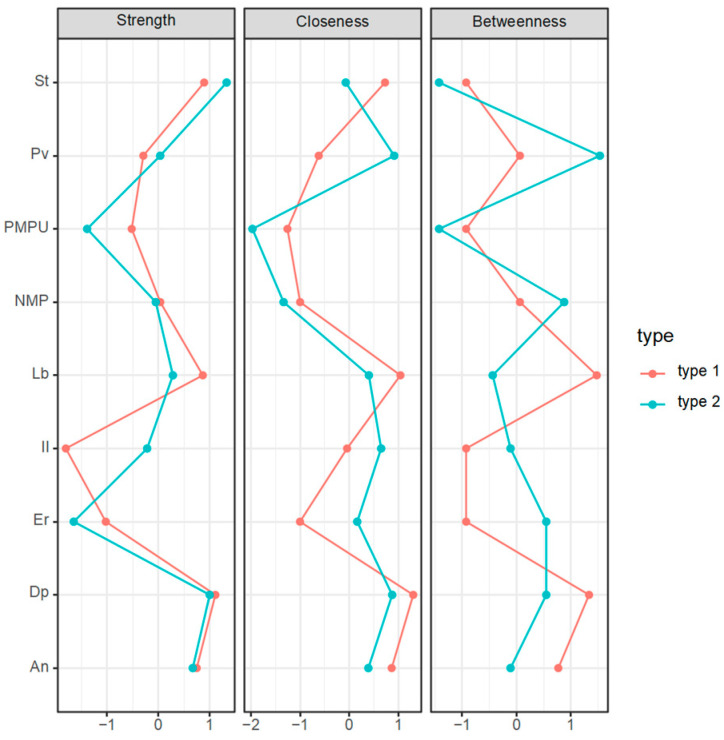
Centrality indices of the factors influencing learning burnout in the networks of different groups.

In order to measure the position of each factor in the network, the centrality indices of each node were further calculated to quantify the position of the nodes in the network. To more intuitively observe whether the relative importance of each node changes between the two networks, they were transformed into standard scores and compared together. The results are shown in Figure 5.

Under the online learning condition depressive mood has the highest strength centrality and closeness centrality as well as a relatively high betweenness centrality, indicating that its association with other variables is closer, and it is located at the center of the network showing that it is a core factor in the network. Interactive learning has the lowest strength centrality, with a weak association with other factors. Stress and learning burnout have a relatively high strength centrality and closeness centrality, having a close association with other variables and being more adjacent. At the same time, learning burnout also has the highest betweenness centrality, indicating that it acts as a role in controlling other connection points, reflecting its mediating and regulating effect.

Under the offline learning conditions depressive mood has a relatively high strength centrality and closeness centrality; however, stress has the highest strength centrality. This is similar to the network under the online learning condition. Learning burnout has no outstanding indicators, while the motivation to pursue value has a relatively high closeness centrality and the highest betweenness centrality.

By comparing the centrality indicators of the two networks, it is found that there are differences. The ranking of the strength centrality indicators of nodes such as problematic mobile phone use and interactive learning has changed, and there are significant changes in the ranking of the closeness centrality and betweenness centrality indicators of the motivation to pursue value and learning burnout nodes. This shows that there are differences in the degree of association between some factors and other factors in the two networks.

#### 3.4.3. Network Stability Test

**Figure 6:**
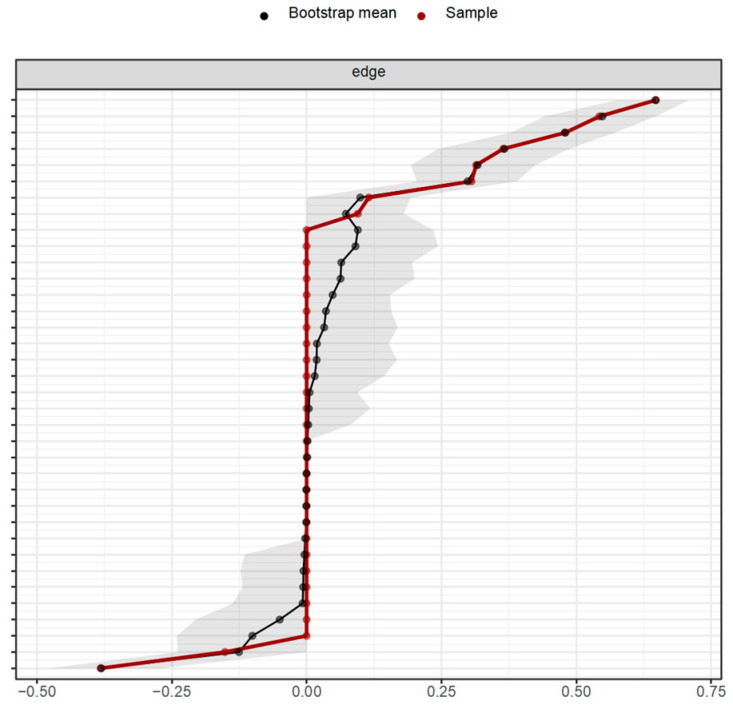
Bootstrap confidence intervals of the weights of the connections in the network of influencing factors of learning burnout under online learning conditions (*n* = 1000).

**Figure 7:**
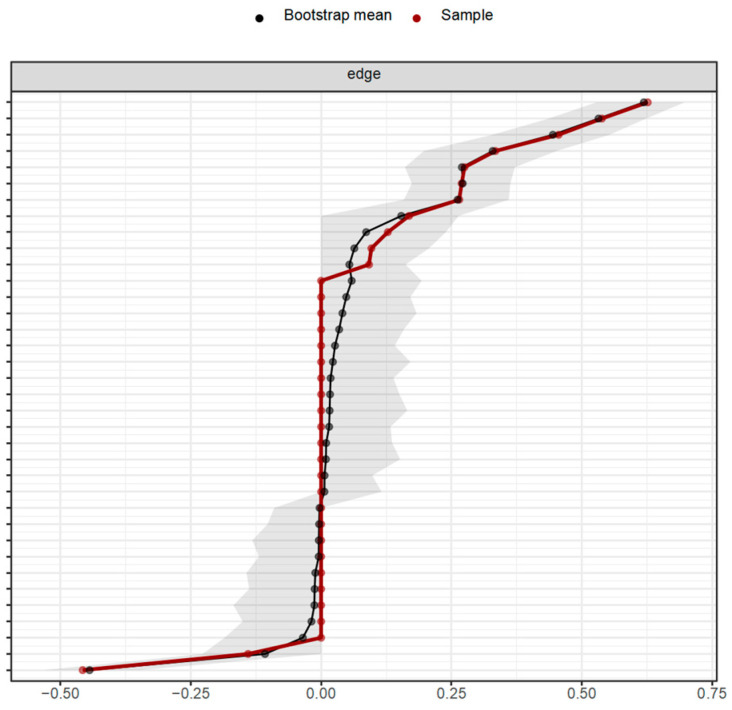
Bootstrap confidence intervals of the weights of the connections in the network of influencing factors of learning burnout under offline learning conditions (*n* = 1000).

Firstly, the bootstrap confidence intervals of each edge were calculated, and the bootstrap method was employed to test the reliability of the connections. Figure 6 and Figure 7, respectively, display the bootstrap confidence intervals of the connection weights in the network of influencing factors of learning burnout under online and offline learning conditions after 1000 iterations of bootstrap resampling. The results indicate that all the previously estimated network connections truly exist, and there are two connections with a negative partial correlation relationship under both online and offline learning conditions.

**Figure 8:**
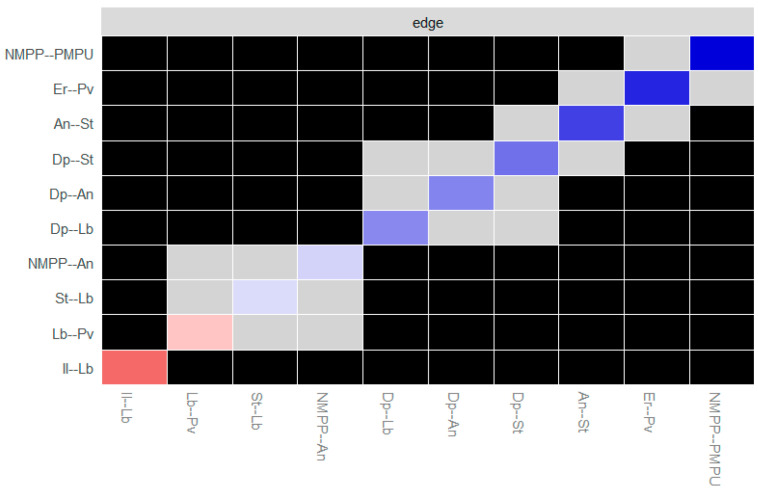
Bootstrap difference test of the weights of the connections in the network of influencing factors of learning burnout under online learning conditions (*n* = 1000, α = 0.05).

**Figure 9:**
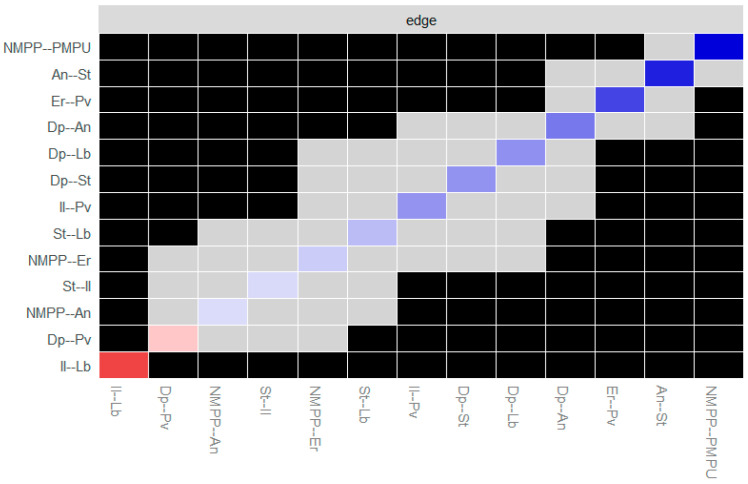
Bootstrap difference test of the weights of the connections in the network of influencing factors of learning burnout under offline learning conditions (*n* = 1000, α = 0.05).

Subsequently, a difference test was conducted on the connections in the network. The results are shown in Figure 8 and Figure 9 (black represents a significant difference between the connections and gray represents no significant difference; the diagonal line indicates the direction and strength of the partial correlation of the connections, where blue represents a positive partial correlation, red represents a negative partial correlation, and the darker the color, the stronger the corresponding partial correlation relationship). It is found that there are significant differences in the two connections with a negative correlation under both the online and offline learning conditions, indicating that there are obvious statistical differences in the relationship strength, nature, or pattern between different node pairs.

**Figure 10:**
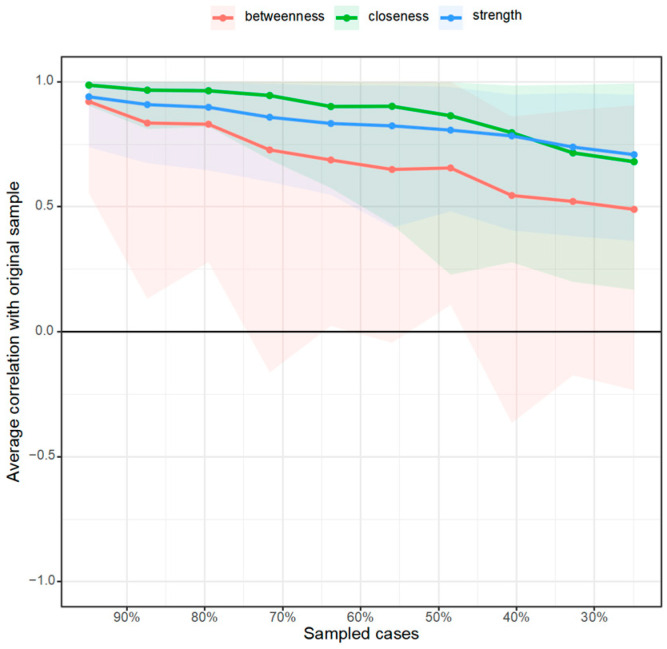
The stability of the centrality indicators of each node in the network of influencing factors of learning burnout under online learning conditions.

**Figure 11:**
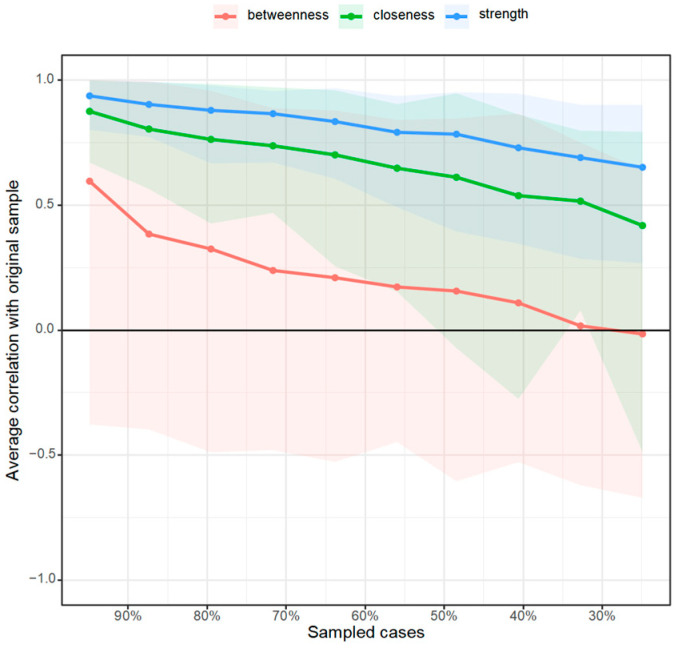
The stability of the centrality indicators of each node in the network of influencing factors of learning burnout under offline learning conditions.

Finally, a stability test of the centrality indicators was carried out. As shown in Figure 10 and Figure 11, by reducing the sample size and checking the centrality indicator sequence to test the stability of the network structure, it was found that under the online learning condition the relevant stability coefficients of strength centrality, closeness centrality, and betweenness centrality were 0.051, 0.126, and 0.000, respectively; moreover, under the offline learning condition the relevant stability coefficients of strength centrality, closeness centrality, and betweenness centrality were 0.283, 0.000, and 0.000, respectively. According to the suggestion of Bringmann et al. (2019), when the relevant stability coefficient is greater than 0.25 the indicator can be considered stable. Different centrality indicators (betweenness centrality, closeness centrality, and strength) behave differently when facing data discarding. In the online learning network betweenness centrality is the most sensitive, and almost complete data is required to ensure the stability of the correlation; closeness centrality has a certain degree of tolerance relatively; and the strength indicator is in an intermediate state, with a certain limit on the proportion of data that can be discarded, but too much data cannot be discarded either. In the offline network the stability coefficient of strength centrality is greater than 0.25, indicating the best stability.

### 3.5. Network Comparison

**Figure 12:**
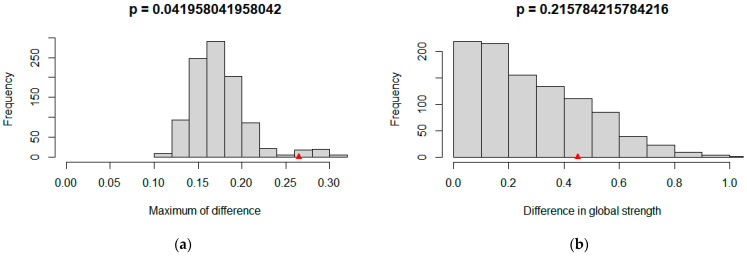
Permutation test of the global invariance of the network of influencing factors of learning burnout in online and offline learning (*n* = 1000). (**a**) Test of network structure invariance; (**b**) test of the overall network strength invariance.

As shown in Figure 12, the results of the Network Comparison Test (NCT) indicate that in terms of global invariance there are significant differences in network structure invariance (*M* = 0.265, *p* = 0.042), while the global strength invariance does not reach a significant level (*S* = 0.449, *p* = 0.216). This means that, at the overall level, although the total connection strengths of the networks of influencing factors of learning burnout under online and offline learning conditions are similar, there are significant structural differences in specific connections. From the perspective of network structure invariance, the significant *p*-value (*p* < 0.05) and the maximum difference (*M* = 0.265) indicate that there are substantial differences in certain edge weights between the two networks (for example, stress and interactive learning, as well as between interactive learning and the motivation for value pursuit), which is also confirmed by local invariance tests. These differences reflect the different mechanisms of action in online and offline environments. In terms of the invariance of the overall network strength, the small difference (*S* = 0.449) and the *p*-value being greater than 0.05 indicate that the total connection strength of the core factors remains consistent across different learning modes.

In terms of local invariance, the study systematically evaluated the connection weights and nodal centralities across the online and offline learning burnout factor networks. Specifically, as shown in Table 3, statistical analyses revealed that the connection weights between stress and interactive learning, as well as between interactive learning and motivation for pursuing value, exhibited significant disparities between the two learning modalities. The weight of the stress and interactive learning connection was notably weaker in the online learning network (*E* = 0.096, *p* < 0.05), and similarly, the interactive learning and motivation for pursuing value connection weight was significantly reduced in the online setting compared to the offline one (*E* = 0.264, *p* < 0.05). In contrast, no significant differences were detected in the weights of other network connections. These findings underscore the differential strength of select factor relationships contingent upon learning environments.

In terms of node centrality, based on the previous results of the stability test of the centrality indices of each node in the two groups this study only conducted a difference test on the strength centrality index. As presented in Table 4, the results showed that there was a significant difference in the strength centrality of interactive learning between the two networks. The strength centrality of interactive learning in the offline learning network was significantly higher than that in the online learning network (*E* = −0.437, *p* < 0.01). Combining with the previous analysis, interactive learning in the offline learning network is associated with more factors, such as the motivation to pursue value and stress, which leads to this result. This further indicates that in terms of the characteristics of local nodes, the degree of association between interactive learning and other factors varies in different learning environments, and interactive learning has a more important position in offline learning.

In conclusion, in terms of global invariance the overall strength of the networks in online learning and offline learning is invariant, yet there are significant differences in the network structures. Regarding local invariance, there are differences between online learning and offline learning. Specifically, there are differences in the connection weights and strength centrality of interactive learning, which have certain implications for understanding the influencing mechanism of learning burnout.

## 4. Discussion

This study found that there are differences in the influencing factors and influencing paths of learning burnout under online and offline learning conditions, and such differences are mainly caused by peer comparison. In an online learning environment, students pay more attention to the realization of personal value. When the learning goals are inconsistent with personal value, it is easy for them to experience learning burnout. In an offline learning environment, however, peer comparison becomes an important factor affecting learning motivation and emotions. When students feel the pressure from their peers, they are prone to having negative emotions, which in turn leads to learning burnout.

### 4.1. Network Structure of Influencing Factors of Learning Burnout and Important Nodes

This study constructs a network structure of influencing factors of learning burnout using network analysis methods, systematically maps the interaction relationships among variables (such as motivation → negative emotions → burnout), and reveals environment-specific pathways.

Under online learning conditions, students are more independent due to the lack of peer comparison and peer pressure. Their motivation for pursuing self-value mainly stems from their own internal drive, manifested as “I want to acquire more knowledge”. In the online context, the motivation for pursuing value directly affects learning burnout. This is consistent with the conclusions of previous studies on the impact of personal goals and motivation on learning burnout (Ryan & Deci, 2000b), further emphasizing the importance of personal values in learning. When students closely link their learning goals with personal values, it can effectively stimulate learning motivation and reduce the probability of learning burnout. For example, self-determination theory emphasizes that intrinsic motivation driven by personal interest and enjoyment is more sustainable and more conducive to learning outcomes than extrinsic motivation. In online learning, students’ intrinsic motivation to acquire knowledge can lead to deeper engagement and lower burnout. Additionally, the absence of distractions and social pressure in online learning allows students to focus on their own learning progress and set goals based on their personal aspirations, rather than through comparisons with others.

In the offline learning environment, there exists peer comparison and peer pressure. The motivation to pursue value not only includes the pursuit of self-value but is also influenced by peer comparison, giving rise to the motivation of “I can’t be worse than others”. Such comparison is likely to trigger negative emotions, which in turn affect learning motivation and lead to a higher level of learning burnout. This result is highly consistent with the social comparison theory (Festinger, 1954). In the process of comparing themselves with others, individuals will generate self-evaluation and emotional responses, thus having an impact on their behaviors and attitudes. The pressure brought about by peer comparison prompts students to develop negative emotions, such as anxiety and feelings of inferiority. These negative emotions will weaken learning motivation and ultimately trigger learning burnout.

Our network analysis extends previous findings by revealing distinct patterns of interaction among psychological factors in online versus offline learning environments. Research shows that in both online and offline learning environments, psychological factors such as depression and stress have a significant positive correlation with learning burnout. These psychological factors not only directly affect learning burnout but also indirectly influence it through other factors. They are intertwined and jointly affect the emergence and development of learning burnout. This indicates that learning burnout is the result of the combined action of multiple factors. It is necessary to comprehensively consider the interactions among various factors and view and understand it from a systematic and integrated perspective (Pekrun et al., 2006). Depressive emotions can cause students to feel down, lose interest in learning, and lack motivation. Stress, on the other hand, can make students feel physically and mentally exhausted, reducing their enthusiasm and confidence in learning. Some research points out that chronic stress can lead to the depletion of students’ psychological resources, making them less able to cope with learning challenges and more susceptible to burnout (Skinner & Pitzer, 2012). In addition, the interaction between depression and burnout can create a vicious cycle, where burnout exacerbates depressive symptoms and vice versa.

Our network analysis extends previous findings by revealing distinct interaction patterns among psychological factors in online and offline learning environments. In the online environment, depression and stress directly predicted burnout (edges: β = 0.28 and β = 0.24), aligning with prior claims of linear associations (Pekrun et al., 2006). In the offline environment, depression not only directly influenced learning burnout (β = 0.25) but also mediated the effect of motivation to pursue value (indirect pathway: β = −0.14 → β = 0.28). This contrasts with the traditional view of motivation as a direct burnout buffer and highlights the critical role of peer comparison in offline settings—a mechanism undervalued in linear models.

In the network structure, key nodes have a significant impact on the overall structure or function of the network. Through calculating the centrality indices of network nodes, this study has identified the key nodes in the network of influencing factors of learning burnout. Under the offline learning condition, the motivation to pursue value is a key node in the network, which indirectly affects learning burnout through depressive emotions. This indicates that in the offline learning environment, students’ learning motivation and values are crucial for preventing learning burnout. This verifies the existing research. Li et al. (2021) pointed out that learning motivation has a positive correlation with self-efficacy, which refers to an individual’s confidence in their ability to complete specific tasks. When students have a high level of learning motivation, they are more likely to believe that they can successfully complete their learning tasks. This belief can significantly reduce learning burnout and increase learning engagement (Zhu, 2016). Therefore, educators should pay attention to students’ pursuit of personal values and guide students to set learning goals that are consistent with their personal values, so as to stimulate students’ learning motivation.

This study also found that psychological factors such as nomophobia and depression–anxiety–stress are also key nodes in the network. These psychological factors not only directly affect learning burnout but also indirectly influence it through other factors.

Based on these findings, in future applications, apart from focusing on key nodes such as emotions and motivation themselves, it is also essential to acknowledge the changes brought by the environment. Online educators should prioritize the management of negative emotions and provide personalized support and interventions (e.g., mindfulness tools) as isolation can amplify their direct harm. Offline educators, meanwhile, must restructure peer interactions (such as through collaborative learning design) to break the pathway of “comparison → depression → burnout” while cultivating intrinsic motivation.

### 4.2. Network Comparison Under the Two Learning Conditions of Online and Offline Learning

By employing a network analytical approach, this study systematically validated how peer comparison-driven mechanisms differentially shape dynamic interaction patterns among learning burnout predictors across online and offline educational contexts.

In terms of global invariance, there are significant differences in the network structures of online learning and offline learning (*M* = 0.265, *p* = 0.042). This indicates that there are different interaction patterns on the whole among the influencing factors of learning burnout in online and offline learning environments. In the online learning environment, the connections between influencing factors are relatively sparse, mainly focusing on personal internal motivation and learning goals. Our network approach identified motivation to pursue value as a key node directly reducing burnout (β = −0.15, *p* < 0.001), suggesting that interventions in online settings should prioritize enhancing intrinsic motivation through personalized goal alignment. In the offline environment, however, the connections between influencing factors are denser, involving more social comparison and emotional responses. Here, motivation to pursue value indirectly reduced burnout via lower depression (β = −0.14 → β = 0.28, *p* < 0.001), highlighting the need to mitigate peer comparison-induced stress through collaborative learning redesign. Meanwhile, the offline environment also involves more social comparison and emotional responses. Although there are significant differences in the overall network structure, when it comes to the relationships of individual nodes and edges it is still necessary to explore local invariance. An analysis of local invariance can help to identify which specific connections or nodes behave differently in the two learning environments, thus revealing the subtle differences in the influencing mechanisms of learning burnout. For example, structural invariance tests revealed that offline learning networks exhibited denser connections around social dynamics (e.g., peer comparison → stress → burnout), whereas online networks emphasized individual agency. This structural shift underscores the importance of context-specific interventions.

Online learning: Leverage technology to provide personalized feedback that reinforces intrinsic motivation.

Offline learning: Implement peer-mentoring programs to reframe social comparison as a tool for growth rather than competition.

In terms of local invariance, there are significant differences in interactive learning under the two learning conditions of online and offline learning. The network revealed weaker connections between interactive learning and other variables online (e.g., stress and motivation), suggesting that interactive learning can provide a certain amount of social interaction and learning support, helping students relieve learning pressure and enhance their enthusiasm and initiative in learning. However, compared with the offline learning environment, the effect of online interactive learning may be restricted by factors such as network technology and interaction methods, and it is unable to fully exert its positive effects.

In the offline learning environment, interactive learning can fully exert its positive effects and is associated with more factors, such as the motivation to pursue value, stress, etc. This is because offline learning provides more realistic social scenarios and more interaction opportunities. Students can better understand and master knowledge through face-to-face communication, cooperative learning, and other means. Notably, interactive learning exhibited higher centrality in offline networks, acting as a bridge between motivation and stress reduction. This implies that fostering peer-led study groups or in-class cooperative tasks could amplify its protective role against burnout. At the same time, it can also enhance their teamwork and social skills (Shu & Gu, 2018). In this environment, the connection between interactive learning and the motivation to pursue value is closer. Through interaction and comparison with others, students can more easily clarify their learning goals and value pursuits, thereby stimulating their learning motivation. There is also a certain correlation between interactive learning and stress. Appropriate stress can prompt students to participate in interactive learning more actively and improve the learning effect. However, excessive stress may cause students to develop anxiety and a psychological tendency to avoid, affecting the effect of interactive learning. For example, educators can incorporate structured peer-collaboration activities (such as small-group problem-solving tasks) into the curriculum. Assigning roles within groups (e.g., facilitator, recorder) can reduce comparison-induced anxiety while promoting accountability and mutual support. To alleviate the additional time burden on teachers, peer-assessment tools or rotating student-led discussions can also be adopted. These approaches leverage student autonomy, reducing direct supervision while maintaining engagement.

In addition, this study found that in the online learning environment, the motivation to pursue value has a more direct and significant impact on learning burnout, while in the offline learning environment, peer comparison has a more prominent influence on learning motivation and emotions. While traditional regression models might overlook these systemic interactions, our network analysis captured nonlinear pathways (e.g., motivation ↔ negative emotions) that inform multi-level interventions. This finding further emphasizes the differences and complexity of the influencing factors of learning burnout in different learning environments. In online learning, students pay more attention to the realization of personal value, and the motivation to pursue value can directly affect learning burnout. When students’ learning motivation aligns with their personal values, they are more likely to maintain a positive learning attitude and reduce the risk of learning burnout. In offline learning, however, peer comparison becomes an important factor influencing learning motivation and emotions. When students compare themselves with their peers, they are prone to generating self-evaluation and emotional responses, which in turn affect their learning motivation and learning burnout. If students feel at a disadvantage in peer comparison, they may experience feelings of frustration and anxiety, which will reduce their learning motivation and increase the likelihood of learning burnout. Conversely, if students receive positive feedback in peer comparison, it may enhance their learning motivation and reduce the level of learning burnout.

Through the comparison of the networks under the two learning conditions of online and offline learning, we have gained a deeper understanding of the mechanisms and differences of the influencing factors of learning burnout in different environments. This provides an important basis for further exploring the formation mechanism of learning burnout and formulating effective intervention strategies.

### 4.3. Prospects and Limitations of the Study

This study has preliminarily verified the new theoretical hypotheses proposed based on the achievements and deficiencies of previous studies and revealed the differences in the influencing factors of learning burnout under online and offline learning conditions, providing a new perspective for understanding the formation mechanism of learning burnout. However, there are still some deficiencies and limitations that need to be improved and refined in future studies.

The stability of the network analysis depends to a large extent on the size of the sample. This study adopted the convenience sampling method, and the representativeness of the sample is limited and the number of samples is small. This may lead to the research results being greatly affected by random variations and accidental factors, making them difficult to replicate, reducing the credibility of the research results, and making it difficult for other researchers to verify them (Barch, 2022). In order to improve the universality and accuracy of the study, it is necessary to expand the sample size and the scope of the sample in future studies, covering students from different regions, majors, and disciplinary backgrounds, so as to make the research results more representative and reliable.

This study only considered the differences in the influencing factors of learning burnout under online and offline learning conditions, and did not involve research in other learning environments, such as the blended learning environment. With the continuous development of educational technology, blended learning environments are becoming increasingly popular. Future research can further explore the impact of different learning environments on the influencing factors of learning burnout, so as to provide a more comprehensive research perspective and offer richer theoretical support for educational practice.

This study mainly adopted the method of questionnaire survey to collect data. Although this method is easy to operate and has high data collection efficiency, it may be subjective and prone to biases. First, the convenience sampling method and limited sample size (N = 293) may restrict the generalizability of findings, particularly for offline learning contexts where unmeasured variables (e.g., classroom norms, instructor immediacy) could uniquely amplify peer comparison effects. Second, while peer comparison was theorized as a key mechanism in offline learning, its indirect measurement (via network pathways like motivation → negative emotions) leaves room for alternative explanations. Future research should incorporate direct measures of peer comparison (e.g., self-report scales) and control for contextual factors (e.g., course design) to isolate its role. Besides this, self-report measures may be subject to social desirability bias, potentially underestimating negative emotions in offline peer comparison scenarios. In future research, other research methods, such as interviews and observations, can be combined to collect more objective and comprehensive data, so as to improve the accuracy and reliability of the research. Interviews can provide an in-depth understanding of students’ learning experiences and inner thoughts, and observations can directly record students’ learning behaviors and interaction situations. Combining these methods with the questionnaire survey can more comprehensively reveal the influencing factors and action mechanisms of learning burnout.

Although the questionnaire survey data used in this study may suffer from recall bias or social desirability effects, the network analysis method can still reveal complex mechanisms that are difficult to capture by traditional linear models through modeling the dynamic relationships between variables (such as indirect effects and mediating paths). For example, the indirect path of ‘motivation → negative emotions → burnout’ (β = −0.14 → β = 0.28) in offline learning indicates that even with limited data granularity, network analysis can deeply analyze the potential role of social comparison.

In psychological network analysis, centrality indices such as closeness centrality and betweenness centrality have certain limitations as tools for measuring the importance of nodes. Centrality indices were initially developed in the context of social networks, and their assumptions and application scenarios may not be fully applicable to the particularities of psychological networks (Bringmann et al., 2019). The nodes in a psychological network represent psychological variables, and the relationships between these variables may not conform to the assumptions of flow and shortest paths on which the centrality indices are based. In addition, the assumptions of the uniqueness and interchangeability of nodes in a psychological network may not hold either. Although some studies have shown that closeness centrality can better describe the influence of end nodes, its application in psychological network analysis still faces challenges. The dynamism and complexity of psychological networks require that centrality indices can adapt to these changes (Elmezain et al., 2021). In this study, the stability of the closeness centrality and betweenness centrality obtained from the sample size is not ideal. Moreover, for psychological networks, closeness centrality and betweenness centrality are not very suitable as indices for measuring the importance of nodes (Bringmann et al., 2019), so they have little impact on this study. Future research can explore centrality indices that are more suitable for psychological network analysis or combine other methods to comprehensively evaluate the importance of nodes, in order to better understand the structure and function of psychological networks.

## 5. Conclusions

(1) This study focuses on the online and offline learning environments and explores the differences in the influencing factors and influencing paths of learning burnout. Building on previous research, it pays attention to the differences between online and offline learning and incorporates the factor of peer comparison. It systematically discusses the influencing factors of learning burnout and the ways to affect learning burnout, providing a comprehensive analysis of the relationships between variables. Finally, a new theoretical model is constructed, which can provide educators and policymakers with a more comprehensive theoretical basis for learning burnout, enabling them to comprehensively consider the advantages and disadvantages of online and offline learning and better take corresponding measures. In the online learning environment, the motivation to pursue value has a direct impact on learning burnout. This indicates that in the online environment of autonomous learning, when the learning goals are closely aligned with personal values then students’ learning motivation is effectively stimulated, thus significantly reducing the level of learning burnout. In the offline learning environment, peer comparison becomes a key factor influencing learning burnout. In a collective learning atmosphere, students’ comparison with their peers triggers negative emotions such as anxiety and feelings of inferiority. These negative emotions weaken learning motivation and ultimately lead to an increase in the level of learning burnout.

(2) By constructing the network structure of the influencing factors of learning burnout, this study reveals the complex relationships among various factors. Psychological factors such as depression and stress are significantly positively correlated with learning burnout in both learning environments, highlighting that learning burnout is the result of the joint action of multiple factors and requires a comprehensive consideration of the interactions among various factors from a systematically integrated perspective. In the network structure, psychological factors such as the motivation to pursue value, nomophobia, and depression–anxiety–stress become key nodes, having an important impact on the overall structure and function of the network. This provides a clear direction for educators, that is, they should pay great attention to students’ pursuit of personal values and actively guide students to set learning goals consistent with their personal values, and at the same time closely monitor students’ mental health conditions and provide necessary psychological support and intervention measures in a timely manner. For example, in online learning settings teachers can guide students to focus on their personal learning progress and goals instead of comparing themselves with others. This can be achieved by offering personalized learning feedback and suggestions. Additionally, teachers can create online learning communities where students are encouraged to share their learning experiences and resources rather than just comparing outcomes. These communities can foster a positive learning environment through mutual support and motivation. For offline learning, teachers can design cooperative learning activities instead of competitive ones. For instance, organizing group projects that emphasize team goals over individual competition can effectively alleviate peer pressure and anxiety while enhancing students’ learning engagement and performance. Moreover, teachers can regularly conduct mental health workshops to help students recognize and manage negative emotions arising from peer comparison. By teaching emotion regulation techniques and positive self-talk methods, these interventions can improve students’ emotional well-being and boost their learning efficiency and overall welfare (Weare & Nind, 2011).

(3) Through the network comparison, this study found that in terms of global invariance the overall strength of the networks of online and offline learning is basically the same, but there are differences in structure. In terms of local invariance, interactive learning shows obvious differences in performance under the two learning conditions. The differences in its connection weights and strength centrality provide important clues for a deeper understanding of the influencing mechanism of learning burnout.

In conclusion, this study has made new breakthroughs in exploring the influencing factors and influencing paths of learning burnout, constructed a more comprehensive theoretical model, and provided educators and policymakers with a richer theoretical basis for learning burnout. Future research should further expand the sample scope to cover students from different regions, majors, and disciplinary backgrounds to improve the universality and accuracy of the research results. It should also explore the impact of different learning environments, such as a blended learning environment, on the influencing factors of learning burnout, combine multiple research methods, comprehensively collect data, and more deeply reveal the formation mechanism of learning burnout. At the same time, attention should be paid to the evaluation of intervention measures and effects to provide more targeted and effective strategies for effectively reducing students’ learning burnout and improving their learning effectiveness and mental health levels.

## Figures and Tables

**Table 1:** Descriptive statistics of learning burnout and its influencing factors under online and offline learning conditions.

	Online Learning	Offline Learning
Nomophobia	77.42 (16.574)	78.82 (15.880)
Problematic Mobile Phone Use	14.12 (3.107)	14.32 (3.216)
Depression	12.31 (4.459)	12.30 (4.463)
Anxiety	12.05 (3.862)	12.12 (3.957)
Stress	14.15 (4.535)	13.76 (4.401)
Interactive Learning	46.51 (8.519)	46.62 (9.037)
Learning Burnout	35.24 (9.301)	35.05 (9.490)
Motivation for Enjoying Relaxation	27.48 (4.958)	27.88 (4.525)
Motivation for Pursuing Value	26.56 (4.331)	26.48 (4.554)

Note. Values are presented as mean (standard deviation).

**Table 2:** Comparison of differences in various influencing factors under the two conditions of online learning and offline learning.

	*t*	*df*	*p*
Nomophobia	−1.623	292	0.106
Problematic Mobile Phone Use	−1.167	292	0.244
Depression	0.065	292	0.948
Anxiety	−0.335	292	0.738
Stress	1.692	292	0.092
Interactive Learning	−0.242	292	0.809
Learning Burnout	0.414	292	0.679
Motivation for Enjoying Relaxation	−1.344	292	0.180
Motivation for Pursuing Value	0.267	292	0.790

**Table 3:** Permutation test of the differences in the weights of some network connections under the environments of online learning and offline learning (*n* = 1000).

Variable 1	Variable 2	*p*	The Difference Value of the Edge Weight
Stress	Interactive Learning	0.039 *	0.096
Interactive Learning	Motivation for Pursuing Value	0.041 *	0.264

Note. * *p* < 0.05.

**Table 4:** Permutation test of the differences in the strength centrality of each node in the network under the conditions of online learning and offline learning (*n* = 1000).

Variable 2	*p*	Strength Difference
Nomophobia	0.621	−0.084
Problematic Mobile Phone Use	0.901	0.021
Depression	0.817	−0.035
Anxiety	0.738	−0.055
Stress	0.530	−0.134
Interactive Learning	0.011 *	−0.437
Learning Burnout	0.865	0.033
Motivation for Enjoying Relaxation	0.733	−0.041
Motivation for Pursuing Value	0.545	−0.166

Note. * *p* < 0.05.

## Data Availability

If data and codes are required then they can be obtained by contacting the corresponding author or the first author.
